# Preclinical comparative study of [^18^F]AlF-PSMA-11 and [^18^F]PSMA-1007 in varying PSMA expressing tumors

**DOI:** 10.1038/s41598-022-20060-7

**Published:** 2022-09-21

**Authors:** Sarah Piron, Jeroen Verhoeven, Jan Courtyn, Ken Kersemans, Benedicte Descamps, Leen Pieters, Anne Vral, Christian Vanhove, Filip De Vos

**Affiliations:** 1 Laboratory for Radiopharmacy, Ghent University, Ghent, Belgium; 2 Department of Diagnostic Sciences, Ghent University, Ghent, Belgium; 3 Department of Medical Imaging, Ghent University Hospital, Ghent, Belgium; 4 IBiTech-MEDISIP, Department of Electronics and Information Systems, Ghent University, Ghent, Belgium; 5 Department of Human Structure and Repair, Ghent University, Ghent, Belgium

**Keywords:** Cancer imaging, Urological cancer

## Abstract

A wide variety of ^18^F-labeled PSMA-targeting PET radiotracers have been developed, including [^18^F]AlF-PSMA-11. As there is only limited data on the comparison with other ^18^F-labeled PSMA PET tracers, a comparative preclinical study between [^18^F]AlF-PSMA-11 and [^18^F]PSMA-1007 was conducted. Mice with varying PSMA expressing tumors (C4-2, 22Rv1 and PC-3, each n = 5) underwent two PET/CT scans with both [^18^F]AlF-PSMA-11 and [^18^F]PSMA-1007. Ten additional mice bearing C4-2 xenografts were subjected to ex vivo biodistribution with either [^18^F]AlF-PSMA-11 (n = 5) or [^18^F]PSMA-1007 (n = 5). Absolute SUV_mean_ and SUV_max_ values were significantly higher for [^18^F]PSMA-1007 scans in both C4-2 tumors (*p* < 0.01) and 22Rv1 tumors (*p* < 0.01). In C4-2 xenograft bearing mice, the tumor-to-organ ratios did not significantly differ between [^18^F]AlF-PSMA-11 and [^18^F]PSMA-1007 for liver, muscle, blood and salivary glands (*p* > 0.05). However, in 22Rv1 xenograft bearing mice, all tumor-to-organ ratios were higher for [^18^F]AlF-PSMA-11 (*p* < 0.01). In healthy organs, [^18^F]PSMA-1007 uptake was higher in the liver, gallbladder, small intestines and glands. Biodistribution data confirmed the increased uptake in the heart, small intestines and liver with [^18^F]PSMA-1007. Absolute tumor uptake was higher with [^18^F]PSMA-1007 in all tumors. Tumor-to-organ ratios did not differ significantly in high PSMA expressing tumors, but were higher for [^18^F]AlF-PSMA-11 in low PSMA expressing tumors. Furthermore, [^18^F]PSMA-1007 showed higher uptake in healthy organs.

## Introduction

The prostate specific membrane antigen (PSMA) is a type II transmembrane protein that is upregulated on prostate cancer cells^1^. Further research showed PSMA to be an excellent target for molecular imaging of prostate carcinoma, which has led to a steep increase in the development of PSMA targeting tracers. Initially, PSMA monoclonal antibodies were introduced targeting either the intracellular domain (7E11 or Capromab Pendetide) or extracellular domain (J591) of PSMA^2,3^. However, monoclonal antibodies have several disadvantageous characteristics for diagnostic purposes, such as a slow tumor uptake and a long circulating half-life. Consequently, the focus of PSMA radiopharmaceutical development has shifted to the development of low molecular weight molecules. These PSMA inhibitors consist usually of a hydrophilic glutamate-urea based pharmacophore coupled to a lipophilic chelator, which target the extracellular zinc pocket and lipophilic pocket of the PSMA protein, respectively. The different affinity of these radiotracers for the PSMA target is determined by the variable amino acid linked to the Glu-urea group and the chelator. Finally, the PSMA inhibitors can be internalized into the cell via clathrin-coated pits^4,5^. [^68^ Ga]PSMA-11 was the first PSMA PET tracer that was widely applied for prostate cancer positron emission tomography/computed tomography (PET/CT) imaging. This PET tracer has demonstrated excellent overall performance in both initial staging (sensitivity of 0.74 (95% CI 0.51–0.89)) and specificity of 0.96 (95% CI 0.85–0.99)) and recurrent prostate cancer (PCa) (detection rate of 0.63 (95% CI, 0.55–0.70) for PSA ≤ 2.0 ng/mL and 0.94 (95% CI, 0.91–0.96) for PSA > 2.0 ng/mL)^6–9^. However, the cyclotron produced fluorine-18 has superior imaging characteristics, including a longer half-life (110 min vs 68 min), a lower positron energy (0.65 MeV vs 1.90 MeV) and a higher positron yield (97% vs 89%)^10^. Therefore, interest in ^18^F-labeled PSMA radiopharmaceuticals increased^11^. As a result, a wide variety of [^18^F]PSMA PET tracers were developed, each with its characteristic advantages and disadvantages in terms of availability, ease of synthesis, binding affinity, pitfalls and biodistribution patterns^12,13^. Out of the extensive pool of [^18^F]PSMA PET tracers, [^18^F]DCFPyL and [^18^F]PSMA-1007 are the most commonly used and their performance has been compared to [^68^Ga]PSMA-11. Studies suggested the non-inferiority of [^18^F]DCFPyL compared to [^68^ Ga]PSMA-11^14,15^, as well as a similar biodistribution pattern^16^. A comparative pilot study between [^18^F]PSMA-1007 and [^68^Ga]PSMA-11 showed that both tracers identified all dominant prostatic lesions, while [^18^F]PSMA-1007 detected some additional low-grade lesions^17^. A meta-analysis by Liu et al. reported a pooled sensitivity and specificity of 0.923 and 0.442 for PSA > 2 ng/mL and 0.832 and 0.277 for PSA ≤ 2 ng/mL, respectively^18^. These results were confirmed by an intra-individual comparative study by Hoberück et al.^19^. The authors reported the exchangeability of both tracers depending on the availability, but highlighted the increased incidence of non-specific bone findings with [^18^F]PSMA-1007. This finding corresponds to the results of a matched-pair comparison by Rauscher et al. which observed a considerably higher number of benign uptake foci with [^18^F]PSMA-1007 (ganglia, 43%; unspecific lymph nodes, 31%; and bone lesions, 24%) compared to [^68^Ga]PSMA-11^20^.

Based on the binding motif of [^68^Ga]PSMA-11, [^18^F]AlF-PSMA-11 was developed. The evaluation in several clinical trials revealed a low radiation burden^21^, good inter-reader reliability^22^ and non-inferiority with [^68^Ga]PSMA-11^23^. However, limited data is available on the comparison of [^18^F]AlF-PSMA-11 with other ^18^F-PSMA PET tracers such as [^18^F]PSMA-1007^24^. Therefore, the aim of this preclinical study was to compare [^18^F]AlF-PSMA-11 to an established ^18^F-PSMA PET tracer. For this purpose, [^18^F]PSMA-1007 was selected as a comparator because of its widely commercial availability^25^. This study includes the intra-individual comparison of mice bearing PCa xenografts with varying PSMA expression as well as an ex vivo biodistribution with [^18^F]AlF-PSMA-11 and [^18^F]PSMA-1007.

## Materials and methods

### Synthesis of PSMA PET tracers

[^18^F]AlF-PSMA-11 was synthesized on a modified SynthraFCHOL synthesis module (Synthra GmbH, Hamburg, Germany) as previously reported^26^. [^18^F]PSMA-1007 was synthesized on an IBA Synthera + platform (IBA, Louvain-la-Neuve, Belgium) as described by Kramer et al.^27^ using a commercially available kit (ABX, Radeberg, Germany).

### Quality control

The radiochemical purity was determined by thin layer chromatography (Alugram RP18-W/UV254 plates (Machery Nagel, Düren, Germany)) using 3:1 v/v acetonitrile in water as mobile phase and resulted in > 96% for [^18^F]AlF-PSMA-11 and > 95% for [^18^F]PSMA-1007. The molar activity (MA) was determined by high performance liquid chromatography (Prevail C18 reversed-phase column, 4.6 × 250 mm, 5 µm, Lokeren, Belgium) and a calibrated dose calibrator, and resulted in a median activity of 187.0 MBq/nmol (range 181.7 – 190.8 MBq/nmol) for [^18^F]AlF-PSMA-11 and 98.2 (range 83.6 – 312.7 MBq/nmol) for [^18^F]PSMA-1007 at the end-of-synthesis.

### Preparation of tumor models

Prostate cancer cells with varying PSMA expression levels were selected: C4-2 (ATCC® CRL-3314, high PSMA expression), 22Rv1 (ATCC® CRL-2505, low PSMA expression) and PC-3 (ATCC® CRL-1435, no PSMA expression). Cells were cultured using RPMI 1640 medium containing 10% FBS, 1% glutamine 200 mM and 1% penicillin/streptomycin (10,000 U/mL) and maintained at 37 °C in 5% CO_2_ in humidified air.

To prepare the cell suspension, the prostate cancer cells were detached, rinsed with FBS-free RPMI 1640 medium and diluted to 5 × 10^6^ cells/100 µL. Four-to-six-week-old male NOD/SCID mice (Janvier, France) were subcutaneously injected at shoulder height with 200 µL 1:1 cell suspension:Matrigel® on either side of each mouse (C4-2, n = 5; 22Rv1, n = 5; PC-3, n = 5). Mice were weekly monitored for tumor growth for 5–6 weeks until tumors reached a diameter between 5 and 10 mm. The study was approved by the Ghent University Ethical Committee on animal experiments (ECD 21/63). All animals were kept and handled according to the European guidelines (Directive 2010/63/EU).

### Small animal PET/CT imaging

All mice received two PET/CT scans with both [^18^F]AlF-PSMA-11 and [^18^F]PSMA-1007 within a timeframe of 4 days (range 1–4 days). All mice were intravenously administered 9.09 ± 0.55 MBq [^18^F]AlF-PSMA-11 with a MA of 61.64 ± 15.83 MBq/nmol and 9.72 ± 0.67 MBq [^18^F]PSMA-1007 with a MA of 53.40 ± 16.44 MBq/nmol. One hour after tracer injection, static total-body PET/CT scans were performed for 15 min, followed by a CT scan for co-registration.

The PET images were acquired in list mode using a small animal PET scanner (β-cube, Molecubes, Ghent, Belgium) with a spatial resolution of 0.85 mm and an axial field-of-view of 13 cm. All PET scans were reconstructed into a 192 × 192 × 384 matrix by an ordered subsets maximization expectation (OSEM) algorithm using 30 iterations and a voxel size of 400 × 400 × 400 µm. High-resolution CT images were acquired using a small animal CT scanner (X-cube, Molecubes, Ghent, Belgium) and iteratively reconstructed with 200 µm voxel size.

### Biodistribution

Ten four-to-six-week-old male NOD/SCID mice (Janvier, France) bearing C4-2 xenografts were subjected to ex vivo biodistribution. All mice received either 2.42 ± 0.09 MBq [^18^F]AlF-PSMA-11 with a MA of 59.78 ± 11.61 MBq/nmol (n = 5) or 2.12 ± 0.11 MBq [^18^F]PSMA-1007 with a MA of 57.44 ± 12.31 MBq/nmol (n = 5). All mice were sacrificed at 1 h post injection (p.i.) and organs including the spleen, intestines, stomach, kidney, bladder, muscle, bone, liver, heart, lungs, brain and testes were removed and collected, as well as a blood sample. All tissues were weighted and measured using a gamma counter (Cobra, Packard, USA).

### Immunohistochemical evaluation

After the last scan, two mice/cell line xenografts were sacrificed and tumors were collected for immunohistochemical (IHC) evaluation of the PSMA expression levels as previously reported^28^. In short, ﻿sections were taken from the center of the tumor sample and stained using Haematoxylin/Eosin, incubated with a primary PSMA antibody (1:400, 2 h, ab133579, Abcam) and counterstained using Haematoxylin (Mayer). Sections were digitally scanned with a virtual scanning microscope (Olympus BX51, Olympus Belgium SA/NV, Berchem, Belgium) at high resolution (20 × magnification).

### Data analysis

Co-registration and analysis of the PET/CT images were performed using PMOD (PMOD Technologies®, Zürich, Switzerland). Volumes of interest (VOIs) were drawn manually for delineating the tumor, kidneys, bladder, salivary, lacrimal and submandibular glands, heart (blood pool), liver, gallbladder, ileum, muscle and bone. Tracer uptake in each VOI was corrected for radioactive decay and residual activity in the syringe. Values were expressed as SUV_mean_ and SUV_max_. Furthermore, tumor-to-organ ratios including tumor-to-liver (TLR), tumor-to-muscle (TMR), tumor-to-blood (TBR) and tumor-to-salivary gland ratio (TSGR) were determined.

Uptake parameters (SUV_mean_, SUV_max_, TLR, TMR, TBR and TSGR) were reported as mean ± SD. The statistical analysis was performed in R^29^ using the Wilcoxon-signed ranks test for the cross-over intra-individual comparison of radiotracer uptake per cell line xenograft. The biodistribution results were compared using the Mann–Whitney U test. The significance level was set to *p* ≤ 0.05.

### Ethics approval and consent to participate

The study was approved by the Ghent University Ethical Committee on animal experiments (ECD 21/63). All animals were kept and handled according to the European guidelines (Directive 2010/63/EU). All methods were carried out in accordance with relevant guidelines and regulations. The study was carried out in compliance with the ARRIVE guidelines.

## Results

### Comparison of [^18^F]AlF-PSMA-11 and [^18^F]PSMA-1007 uptake in varying PSMA expressing xenografts

**Figure 1:**
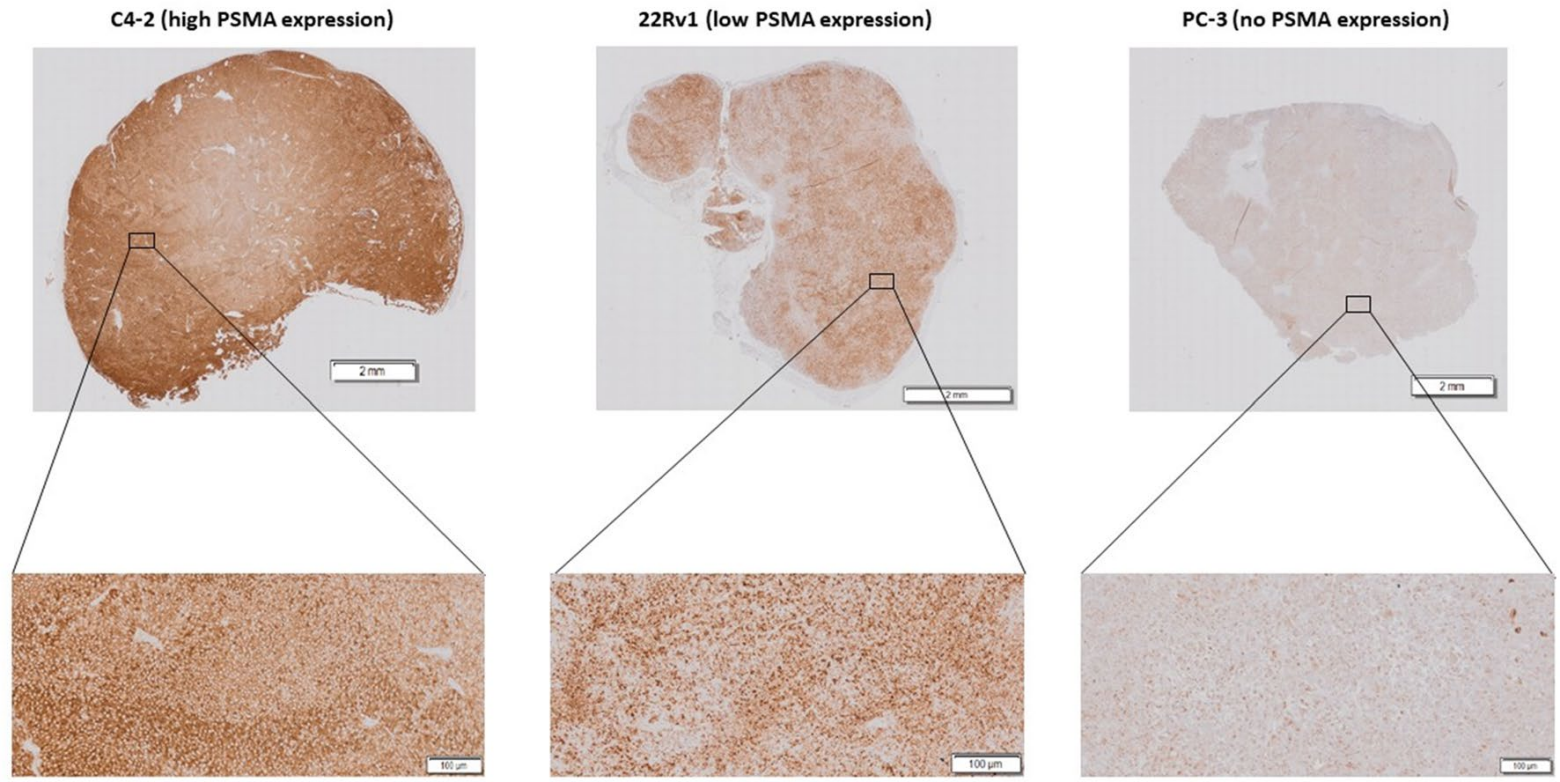
Immunohistochemical staining of tumor tissues of C4-2, 22Rv1 and PC-3 confirming the differences in PSMA expression.

Mice were inoculated with either a high PMSA expressing cell line (C4-2), a low PSMA expressing cell line (22Rv1) or a PSMA negative cell line (PC-3). Immunohistochemical staining was applied on tumor tissues after the last scan and confirmed the differences in PSMA expression levels (Fig. 1).

**Figure 2:**
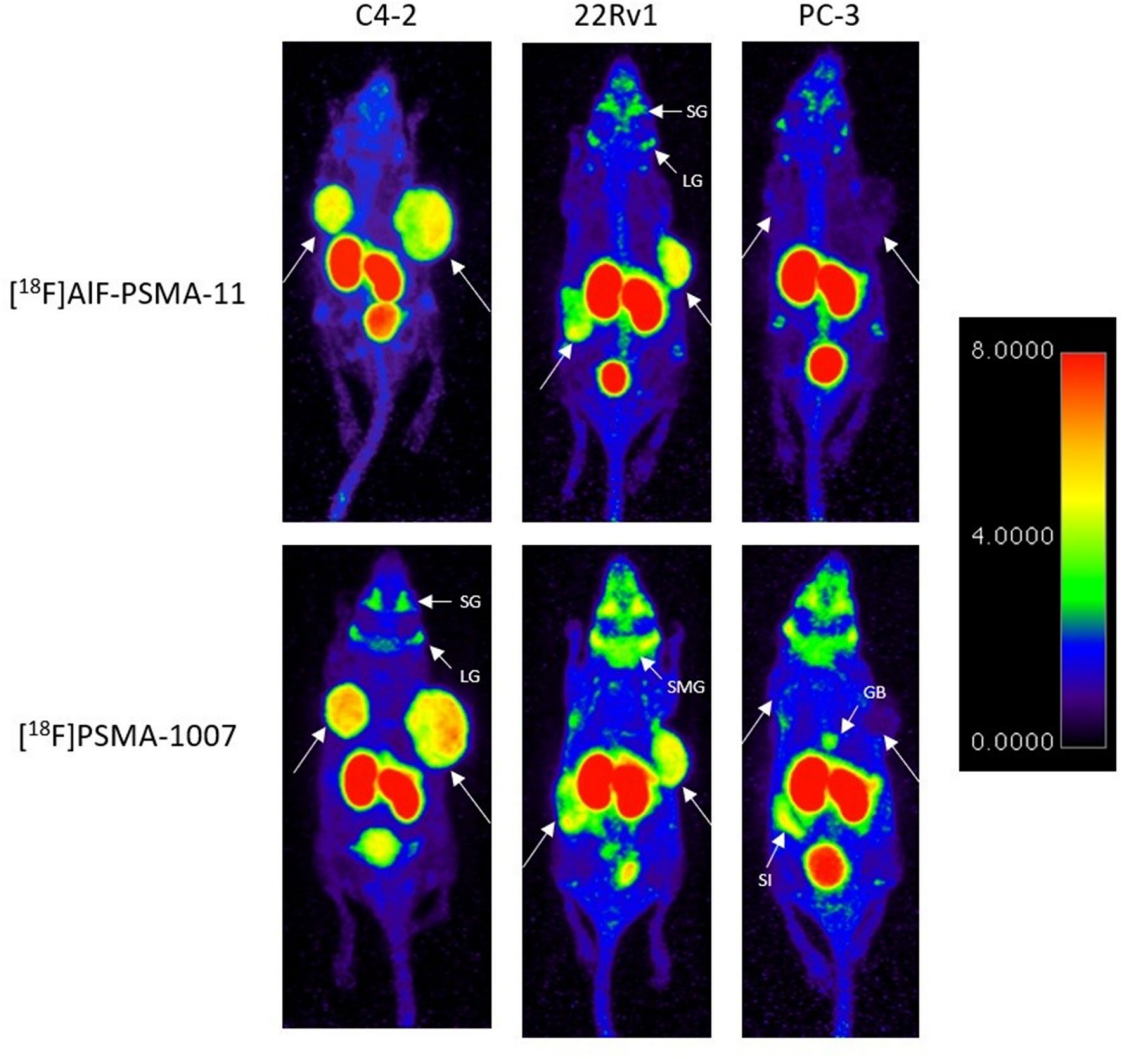
Representative images of mice with either C4-2 (high PSMA expression), 22Rv1 (medium PSMA expression) and PC-3 (no PSMA expression) xenografts. All mice underwent PET/CT imaging 60 min after the administration of [^18^F]AlF-PSMA-11 and [^18^F]PSMA-1007. SG = salivary gland, LG = lacrimal gland, SMG = submandibular gland, GB = gallbladder, SI = small intestines. Color maps were generated using Horos v4.0.0, https://horosproject.org/.

Each mouse underwent a static [^18^F]AlF-PSMA-11 and [^18^F]PSMA-1007 PET scan for 15 min after an uptake period of 1 h within a median time window of 4 days (range 1–4 days). Representative images of one mouse/cell line xenograft are presented in Fig. 2.

C4-2 tumors (high PSMA expression) and 22Rv1 tumors (low PSMA expression) were clearly visible with both PSMA tracers, but uptake in the latter was less intense. No activity uptake was observed in PC-3 tumors. With both ^18^F-PSMA tracers, activity uptake was observed in healthy organs including salivary and lacrimal glands, kidneys and bladder. For [^18^F]PSMA-1007, additional activity uptake was observed in the submandibular glands, intestines and gallbladder. The activity uptake in the glands seems to increase from high PSMA expression (C4-2) to no PSMA expression PC-3).

**Figure 3:**
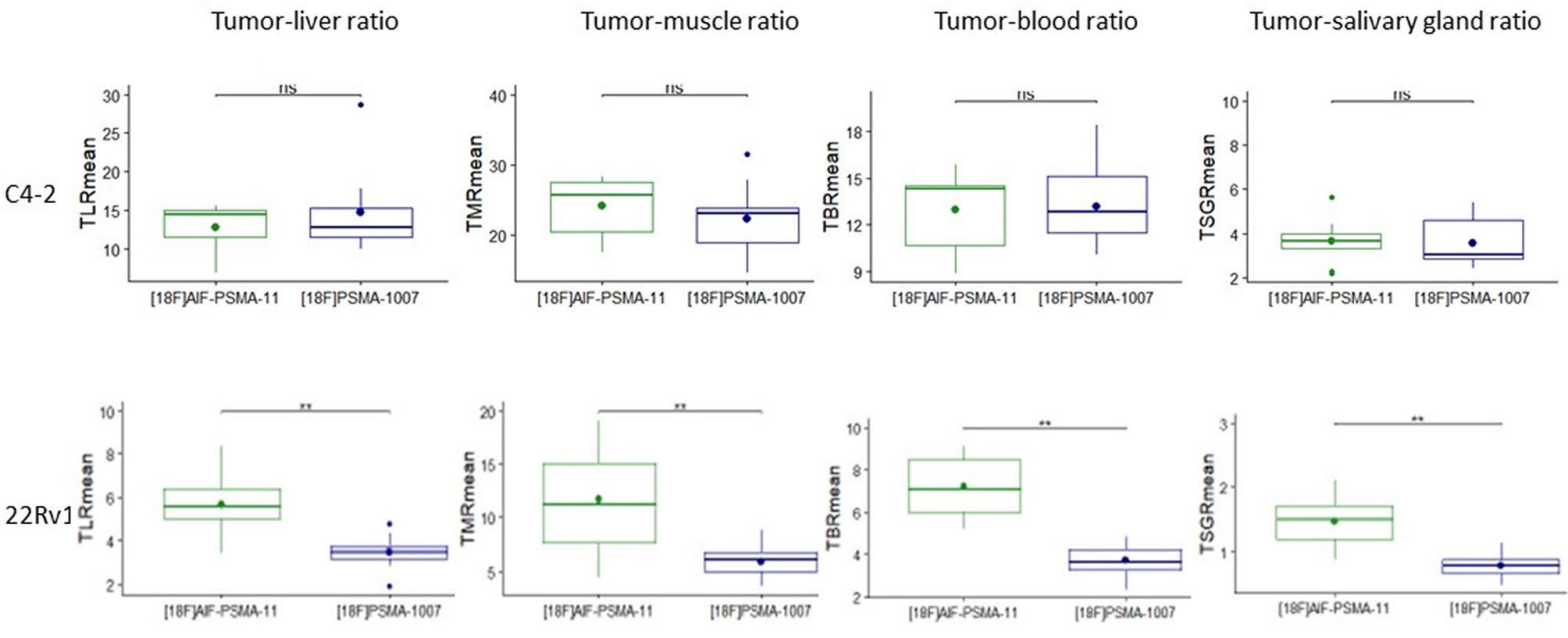
TLR_mean_ (liver), TMR_mean_ (muscle), TBR_mean_ (blood) and TSGR_mean_ (salivary gland) for [^18^F]AlF-PSMA-11 and [^18^F]PSMA-1007 uptake in C4-2 and 22Rv1 xenograft bearing mice. The dot presents the mean, the horizontal bar presents the median value. Ns = not significant, * = p < 0.05, ** = p < 0.01.

Absolute SUV_mean_ and SUV_max_ values in both C4-2 tumors (high PSMA expression) and 22Rv1 tumors (low PSMA expression) were significantly higher for [^18^F]PSMA-1007 scans (*p* < 0.01) (Table 1). In PC-3 tumors (no PSMA expression), the activity uptake was similar between both PSMA PET tracers. In C4-2 xenograft bearing mice, the tumor-to-organ ratios did not significantly differ between [^18^F]AlF-PSMA-11 and [^18^F]PSMA-1007 for ratios including TLR_mean_ (liver) (12.68 ± 3.06 vs 14.76 ± 5.82 respectively, *p* = 0.2), TMR_mean_ (muscle) (24.07 ± 4.14 vs 22.22 ± 5.24, respectively, *p* = 0.91), TBR_mean_ (blood) (12.96 ± 2.61 vs 13.20 ± 2.75, respectively, *p* = 0.73) and TSGR_mean_ (salivary glands) (3.68 ± 1.05 vs 3.58 ± 1.08, respectively, *p* = 0.65) (Fig. 3). Similar trends were found for maximum tumor-to-organ ratios (Supplementary Data Figure S1). However, in 22Rv1 xenograft bearing mice, all tumor-to-organ ratios were higher for [^18^F]AlF-PSMA-11, including TLR_mean_ (liver) (5.70 ± 1.46 vs 3.45 ± 0.79 respectively, *p* = 0.002), TMR_mean_ (muscle) (11.68 ± 5.06 vs 5.92 ± 1.58, respectively, *p* = 0.002), TBR_mean_ (blood) (7.19 ± 1.47 vs 3.72 ± 0.76, respectively, *p* = 0.002) and TSGR_mean_ (salivary glands) (1.48 ± 0.39 vs 0.78 ± 0.18, respectively, *p* = 0.002). Similar trends were found for maximum tumor-to-organ ratios (Supplementary Data Figure S2).

**Table 1:** SUV_mean_ and SUV_max_ values for [^18^F]AlF-PSMA-11 and [^18^F]PSMA-1007 uptake in C4-2 (high PSMA expression), 22Rv1 (low PSMA expression) and PC-3 (no PSMA expression). Values are expressed as mean ± SD. SUV = Standardized Uptake Value.

	SUV_mean_			SUV_max_		
	[^18^F]AlF-PSMA-11	[^18^F]PSMA-1007	*p*	[^18^F]AlF-PSMA-11	[^18^F]PSMA-1007	*p*
C4-2	1.65 ± 0.33	2.59 ± 0.37	**0.0039**	3.58 ± 1.00	5.52 ± 1.00	**0.0039**
22Rv1	0.64 ± 0.23	0.88 ± 0.21	**0.002**	1.58 ± 0.59	2.35 ± 0.80	**0.0059**
PC-3	0.24 ± 0.08	0.22 ± 0.05	0.38	0.50 ± 0.15	0.61 ± 0.20	0.31

Significant values are in bold.

### Comparison of [^18^F]AlF-PSMA-11 and [^18^F]PSMA-1007 uptake in healthy organs

**Figure 4:**
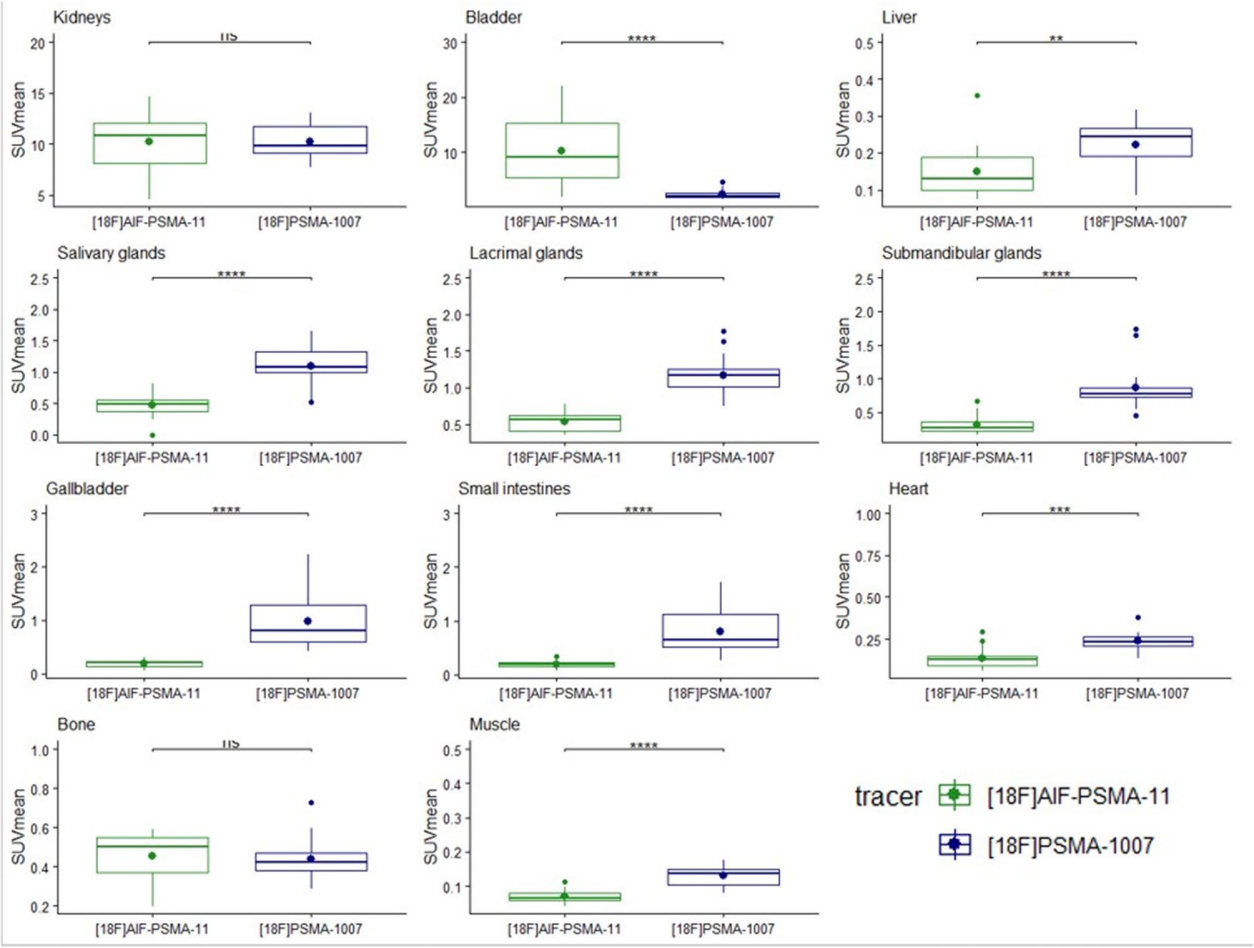
SUV_mean_ for [^18^F]AlF-PSMA-11 and [^18^F]PSMA-1007 uptake in healthy organs: kidneys, bladder, liver, salivary glands, lacrimal glands, submandibular glands, gallbladder, small intestines, heart and bone. The dot presents the mean, the horizontal bar presents the median value. Ns = not significant, * = p < 0.05, ** = p < 0.01, *** = p < 0.001, **** = p < 0.0001.

Comparison of SUV_mean_ values in organs suggests an overall higher activity uptake of [^18^F]PSMA-1007 in background tissues (Fig. 4). Overall, [^18^F]AlF-PSMA-11 showed a higher degree of urinary clearance because of higher uptake in the bladder. However, the variability of the uptake values is high because of urination of the mice after injection and before the scan. Compared to [^18^F]AlF-PSMA-11, a higher amount of [^18^F]PSMA-1007 was detected in the gallbladder (SUV_mean_ of 0.97 ± 0.51 vs 0.19 ± 0.06 respectively, *p* < 0.0001) and the liver (SUV_mean_ of 0.22 ± 0.07 vs 0.15 ± 0.07 respectively, *p* < 0.01), although the absolute uptake in the liver was relatively low for both PSMA tracers. No statistically significant difference could be observed in the kidneys. All glands (salivary, lacrimal and submandibular) demonstrated higher [^18^F]PSMA-1007 activity uptake compared to [^18^F]AlF-PSMA-11, as well as the activity in the heart (blood pool) (SUV_mean_ 0.24 ± 0.06 vs 0.14 ± 0.07 respectively, *p* < 0.001). Increased uptake of [^18^F]PSMA-1007 was also observed in the intestines with mean SUV_max_ values of 1.40 ± 0.89 with a maximum SUV_max_ value up to 3.33. Finally, no statistically significant difference could be observed in bone (spine) for [^18^F]PSMA-1007 and [^18^F]AlF-PSMA-11 (SUV_mean_ 0.44 ± 0.11 vs 0.45 ± 0.11 respectively, *p* = 0.46).

### Ex vivo comparison of [^18^F]AlF-PSMA-11 and [^18^F]PSMA-1007 uptake in healthy organs

The tracer uptake in healthy organs was more thoroughly investigated by ex vivo biodistribution. Values are presented as percentage injected dose normalized for the weight of the organs (Table 2). Biodistribution data confirmed the increased uptake in the heart, small intestines and liver with [^18^F]PSMA-1007. Although the uptake in the brain is statistically significant, the absolute values are insignificantly small. Furthermore, no significant difference could be found in the bladder, blood, bone (sternum), large intestines, lungs, spleen, stomach and testes.

**Table 2:** Biodistribution data of [^18^F]AlF-PSMA-11 and [^18^F]PSMA-1007 of healthy organs. The values represent the percentage injected dose per gram tissue weight 1 h after injection. %ID/g = percentage injected dose per gram.

Organ	%ID/g		
	[^18^F]AlF-PSMA-11	[^18^F]PSMA-1007	*p*
Bladder	2.41 ± 0.90	3.13 ± 1.39	0.42
Blood*	0.57 ± 0.15	1.00 ± 0.06	0.016
Bone	1.16 ± 0.34	0.89 ± 0.05	0.42
Brain*	0.05 ± 0.01	0.10 ± 0.02	0.016
Heart*	0.64 ± 0.18	1.33 ± 0.29	0.016
Kidneys**	111 ± 30.10	47.7 ± 12.2	0.0079
Large intestines	0.28 ± 0.13	0.36 ± 0.18	0.55
Small intestines*	0.26 ± 0.08	0.94 ± 0.43	0.016
Liver**	0.39 ± 0.09	1.08 ± 0.38	0.0079
Lungs	1.69 ± 0.31	1.88 ± 0.63	0.84
Spleen	15.28 ± 4.27	18.20 ± 9.2	0.9
Stomach	0.37 ± 0.07	0.46 ± 0.18	0.15
Testes	0.79 ± 0.19	0.68 ± 0.12	0.31

## Discussion

Currently, there are several PSMA targeting PET tracers used in clinical practice. Several comparative studies between PSMA PET tracers have been conducted^15,17,20,30–34^. Although most comparative studies have suggested the overall comparable performance of these PET tracers, there are other factors to consider. [^18^F]PSMA-1007 has a more complex synthesis route including a critical [^18^F]fluoride azeotropic drying step whereas the synthesis of [^18^F]AlF-PSMA-11 can be performed in aqueous conditions. Where the PSMA-11 precursor is widely available for purchasing and in-house implementation, [^18^F]PSMA-1007 is commercially available as cassettes^25^. Several preclinical comparative studies between [^18^F]AlF-PSMA-11 and [^68^Ga]PSMA-11 have been conducted^35,36^, but data on the comparison with other ^18^F-labeled PSMA PET tracers is rather limited.

PET images of both C4-2 and 22Rv1 xenograft bearing mice showed higher absolute uptake values for [^18^F]PSMA-1007 compared to [^18^F]AlF-PSMA-11, although the difference for the low PSMA expression 22Rv1 tumors is less pronounced. The reported uptake values of [^18^F]PSMA-1007 for C4-2 tumors (comparable PSMA expression compared to LNCaP^37^) seem to be in line with previously reported values by Soeda et al. for LNCaP tumors (SUV_mean_ of 1.81 ± 0.57 and SUV_max_ of 5.4 ± 2.6 versus SUV_mean_ of 2.59 ± 0.37 versus SUV_max_ of 5.5 ± 1.0 in this study)^38^.

Although the absolute uptake values were higher for [^18^F]PSMA-1007, the tumor-to organ ratios show no significant difference between both tracers in C4-2 tumors (high PSMA expression). Furthermore, the tumor-to-organ ratios for [^18^F]AlF-PSMA-11 were significantly higher in 22Rv1 tumors (low PSMA expression). This could be explained by the higher uptake of [^18^F]PSMA-1007 in healthy tissues such as the liver, heart, and glands as well as the less pronounced absolute difference in tumor uptake between both PSMA tracers in low PSMA expressing tumors. These results suggest that [^18^F]AlF-PSMA-11 can be useful for the detection of lesions in proximity to organs with higher [^18^F]PSMA-1007 uptake such as the gallbladder, liver, heart (blood pool) and small intestines. However, the reverse also applies for the bladder and kidneys, as [^18^F]AlF-PSMA-11 is primarily renally excreted. Prostate cancer tumors with a low PSMA expression have been shown to be a negative prognostic factor for overall survival. A study by Seifert et al. investigated the correlation between PSMA expression and overall survival in patients who underwent [^177^Lu]PSMA therapy. Patients with low PSMA expressing lesions were reported to have a shorter survival (7.9 months) compared to patients without low PSMA expressing lesions (21.3 months, *p* = 0.003). Whether this can be attributed to reduced efficacy of [^177^Lu]PSMA therapy or to dedifferentiated and more aggressive tumor phenotypes, remains unclear^39^. Nevertheless, it remains important to detect the presence of low PSMA expressing metastases to aid in the prognostication and decision making process regarding treatment plan. The higher tumor-to-organ ratios for [^18^F]AlF-PSMA-11 compared to [^18^F]PSMA-1007 may therefore be beneficial for the detection of low PSMA expressing tumors. This will mostly depend on the location of the lesion, as the absolute tumor uptake remains higher with [^18^F]PSMA-1007. Furthermore, the detection of non-prostatic tumors is a potential application these are mostly characterized by a low PSMA expression^40^.

Although the tumor-to-muscle ratio seems to be significantly higher for [^18^F]AlF-PSMA-11, the absolute uptake values in the muscle are relatively low (0.13 ± 0.03 for [^18^F]PSMA-1007 compared to 0.07 ± 0.02 for [^18^F]AlF-PSMA-11), which renders this parameter clinically less relevant. Images in Fig. 2 showed higher activity uptake in glands of mice with PSMA negative tumors compared to high PSMA expressing tumors. This could be attributed to more PSMA binding to the tumor, reducing the amount of PSMA tracer left to bind aspecifically.

As the molar activity has a large influence on the tumor uptake, both radiotracers were administered in comparable MA values (61.6 ± 15.8 MBq/nmol for [^18^F]AlF-PSMA-11 and 53.4 ± 16.4 MBq/nmol for [^18^F]PSMA-1007). Although [^18^F]PSMA-1007 has been reported to achieve molar activities up to 1000 MBq/nmol^27,38,41^, the study by Soeda et al. showed no significant difference in tumor uptake values between 1000 and 100 MBq/nmol. However, at these higher MA, the uptake in the salivary glands increased significantly^38^.

Potential defluorination leading to benign bone uptake is always one of the major key points when considering the use of [^18^F]AlF-PSMA-11. Although some studies have reported increased bone uptake with [^18^F]AlF-PSMA-11^24,35^, we did not observe this in our study, both in the imaging and biodistribution experiments. This can possibly be explained by the applied storage conditions. As [^18^F]AlF-PSMA-11 is less stable at room temperature, the activity vial was cooled until administration of the tracer. The same procedure is maintained when used in our clinical routine and this seems to significantly reduce the defluorination process.

Several organs were difficult to delineate on PET images. For example, the spleen is located too closely to the kidneys to be accurately delineated, and it is difficult to distinguish between the large and small intestine on imaging. Therefore, additional biodistribution data of healthy organs was collected. Although PET images demonstrated increased uptake in the bladder of [^18^F]AlF-PSMA-11, biodistribution data did not show a significant difference. This can be explained as the bladder was emptied in the biodistribution experiment while mice were imaged with a full bladder. Therefore, the data suggests that the increased uptake is due to the primarily urinary excretion of [^18^F]AlF-PSMA-11. PET images showed increased uptake spots in the intestines with [^18^F]PSMA-1007. Biodistribution data revealed a statistically significant higher uptake in the small intestines (0.94 ± 0.43%ID/g for [^18^F]PSMA-1007 vs 0.26 ± 0.08%ID/g for [^18^F]AlF-PSMA-11, *p* = 0.016). These results are consistent with the study by Soeda et al. who also reported increased uptake in the small intestines^38^. Overall, [^18^F]PSMA-1007 seems to accumulate more in healthy organs that express PSMA to a low extent (small intestines, salivary and lacrimal glands, liver and spleen). This might be caused by a higher affinity of [^18^F]PSMA-1007 compared to [^68^Ga]PSMA-11^42,43^, which has been shown to have a similar affinity to [^18^F]AlF-PSMA-11^36^. Although this probably leads to the superior tumor uptake, this also causes more aspecific uptake in non-tumor tissue, increasing the background activity. A matched-pair comparative study evaluating the frequency of benign uptake for [^18^F]PSMA-1007 and [^68^Ga]PSMA-11, suggested that five times more lesions (245 out of 369 PSMA-uptake positive lesions) could be attributed to a benign origin for [^18^F]PSMA-1007 compared to 52 of 178 PSMA-uptake positive lesions for [^68^Ga]PSMA-11^20^. This difference was attributed to either the higher affinity of [^18^F]PSMA-1007 and the superior spatial resolution of fluorine-18. An overview of the strengths and weaknesses of both radiotracers is given in Table 3.

**Table 3:** Overview of the strengths and weaknesses of [^18^F]AlF-PSMA-11 and [^18^F]PSMA-1007.

	[^18^F]AlF-PSMA-11	[^18^F]PSMA-1007
Strenghts	Simple, straightforward synthesis in aqueous conditions	Commercially available cassette system
	Precursor is freely available	Stable at room temperature
	High PSMA affinity leads to specific tumor uptake and low non-tumor uptake in healthy organs	Very high PSMA affinity leads to specific and high absolute tumor uptake
	Low uptake in healthy organs results in high tumor-to-organ ratios	Low urinary clearance causes less interference for the detection of local recurrent disease
Weaknesses	No commercially available synthesis platform	More complex synthesis with azeotropic drying step
	End product should be cooled to ensure the stability over a longer time period	Very high PSMA affinity leads to uptake in non-tumor tissues including salivary, lacrimal and submandibular glands, gall bladder, small intestines and spleen
	Lower absolute tumor uptake	High uptake in background tissues results in lower tumor-to-organ ratios
	Renal clearance leads to high activity in the bladder, which could interfere with the detection of local recurrent disease	Hepatobiliary clearance leads to higher tracer uptake in de liver, potentially obscuring liver lesions

The major limitation to this study is the difference in PSMA expressing tissues between mice and humans. Because of the high PSMA expression in murine kidneys, it is possible that kidney activity in humans may give different results. However, imaging and biodistribution data concerning the bladder and liver suggests that the primary excretion pathway for [^18^F]PSMA-1007 (hepatobiliary clearance) and [^18^F]AlF-PSMA-11 (urinary clearance) is comparable between mice and humans.

## Conclusion

Both [^18^F]AlF-PSMA-11 and [^18^F]PSMA-1007 demonstrated good image quality. High and low PSMA expressing tumors demonstrated higher absolute tumor uptake with [^18^F]PSMA-1007, but tumor-to-organ ratios did not differ significantly and were higher with [^18^F]AlF-PSMA-11 in low PSMA expressing xenograft bearing mice. This may be attributed to increased [^18^F]PSMA-1007 uptake in healthy organs such as the liver, heart and salivary glands but also in the gallbladder and small intestines. Whether these preclinical observations will result in clinically relevant differences between both radiotracers should be further investigated.

## Supplementary Information

Supplementary Information.

## Data Availability

The datasets generated during the current study are available from the corresponding author on reasonable request.

## Abbreviations

CT: Computed tomography,
IHC: Immunohistochemistry
OSEM: Ordered subsets maximization expectation
p.i.: Post injection
PET: Positron emission tomography
PSMA: Prostate specific membrane antigen
SUV: Standardized uptake value
TBR: Tumor-to-blood ratio
TLR: Tumor-to-liver ratio
TMR: Tumor-to-muscle ratio
TSGR: Tumor-to-salivary gland ratio
VOI: Volume of interest
