# Supplementary material for: Preclinical comparative study of [18F]AlF-PSMA-11 and [18F]PSMA-1007 in varying PSMA expressing tumors
Source: Sci Rep. 2022 Sep 21;12:15744. doi: 10.1038/s41598-022-20060-7 (PMC9492661; doi:10.1038/s41598-022-20060-7)

Supplementary data: Preclinical comparative study of [^{18}F]AlF-PSMA-11 and [^{18}F]PSMA-1007 in varying PSMA expressing tumors

Figure S1: TLR_{max} (liver), TMR_{max} (muscle), TBR_{max} (blood) and $TSGR_{max}$ for [^{18}F]AlF-PSMA-11 and [^{18}F]PSMA-1007 uptake in C4-2 xenograft bearing mice. The dot presents the mean, the horizontal bar presents the median value. Ns = not significant, * = p<0.05, ** = p<0.01.

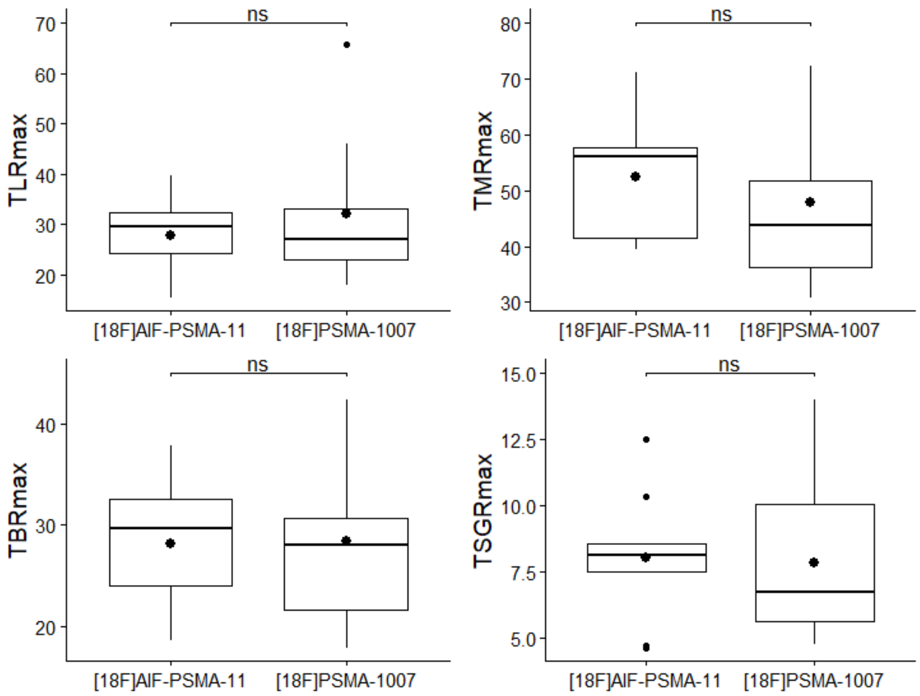

Figure S2: TLR_{max} (liver), TMR_{max} (muscle), TBR_{max} (blood) and $TSGR_{max}$ for [^{18}F]AlF-PSMA-11 and [^{18}F]PSMA-1007 uptake in 22Rv1 xenograft bearing mice. The dot presents the mean, the horizontal bar presents the median value. Ns = not significant, * = p<0.05, ** = p<0.01

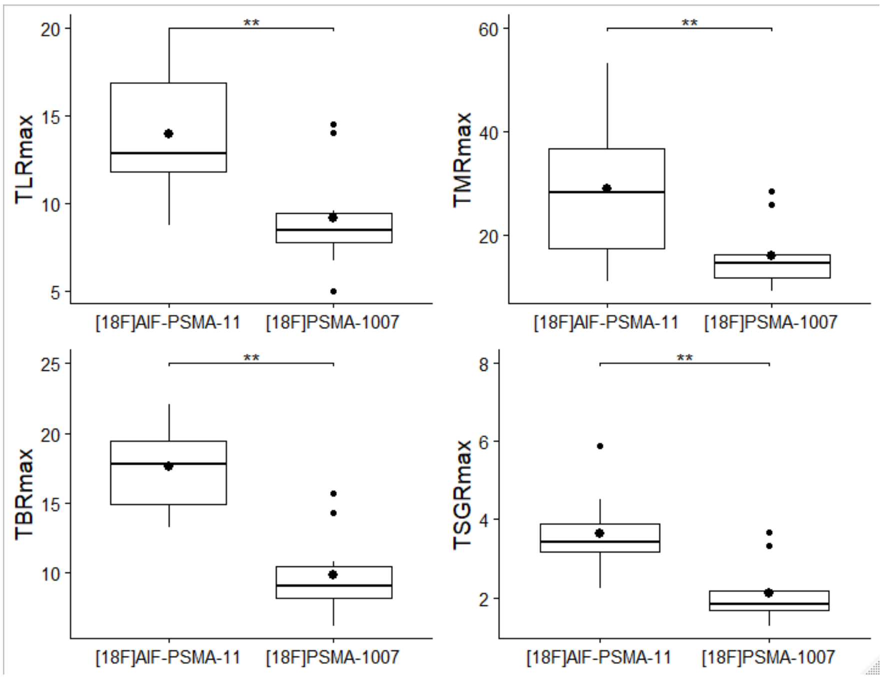

Supplement: Supplementary file 1 — Supplementary Information. [file 41598_2022_20060_MOESM1_ESM.pdf]
